# Supplementary material for: Correlates of emotional violence against children in Rwanda: Findings from a cross-sectional national survey
Source: PLoS One. 2023 Jun 13;18(6):e0286788. doi: 10.1371/journal.pone.0286788 (PMC10263359; doi:10.1371/journal.pone.0286788)
Supplement: Attachment — Submitted filename: Response to Reviewers.docx [file pone.0286788.s002.docx]

I am very thankful to the reviewers who have taken their time and put their efforts into reviewing my manuscripts. I have no doubt that their comments have been very critical in improving the quality and content of my manuscript. May God Bless you all you. Below is also my feedback on comments on the initial submission to my manuscript.

| **Reviewer comments** | **Authors response** |
| --- | --- |
| **Journal Requirements:** | |
| 1. Please ensure that your manuscript meets PLOS ONE's style requirements, including those for file naming. | **Comment accepted and addressed:**   - The manuscript has been updated and formatted according to PLOS ONE's style requirements and file naming. |
| 2. Please provide additional details regarding participant consent. In the ethics statement in the Methods and online submission information, please ensure that you have specified (1) whether consent was informed and (2) what type you obtained (for instance, written or verbal, and if verbal, how it was documented and witnessed). If your study included minors, state whether you obtained consent from parents or guardians. If the need for consent was waived by the ethics committee, please include this information.   If you are reporting a retrospective study of medical records or archived samples, please ensure that you have discussed whether all data were fully anonymized before you accessed them and/or whether the IRB or ethics committee waived the requirement for informed consent. If patients provided informed written consent to have data from their medical records used in research, please include this information. | **Comment accepted and addressed:**   - Under the Methods section, in the sub-section talking about data, I have included a paragraph with additional details on ethical considerations that were applied by the survey to collect data. |
| 3. We note you have included a table to which you do not refer in the text of your manuscript. Please ensure that you refer to Tables 1,2 and 3 in your text; if accepted, production will need this reference to link the reader to the Table. | **Comment accepted and addressed:**   - Tables 1, 2 and 3 are now referred to before inclusion in the text of the updated manuscript. |
| **Additional Editor Comments** | |
| Aim: This study used data from the Rwanda Survey to examine patterns of emotional violence (EV) and associated, and to describe the profile of children who were emotionally abused.  Says “All data are within the manuscript and supporting documents”. But no supporting documents found. | The datasets used and analyses performed for this study are available from the corresponding author (**Alypio Nyandwi; Email:** [**nalypio@gmail.com**](mailto:nalypio@gmail.com)) and can be obtained on reasonable request. |
| Agree with authors view that “identification of emotional violence largely depends on the dialogue with the victim who, through his narrated experiences, can disclose about his experiences of emotional violence. Consequently, emotional violence tends to be hidden and might be underestimated or given less attention than child physical and sexual abuse”. Readers need at least briefly know how these were tackled in methods, even if they were detailed in previous publications. Specifically, what were the measures taken to mitigate possible information bias in survey data collection from children? In other words, can they remain as limitations or points to consider in future research? | **Comment accepted and addressed.**   - I have added a few paragraphs clarifying and describing the status of emotional violence globally. I also talked about the use of Violence against Children and Youth Surveys (VACS) as one tool to collect national data on violence against children, including emotional violence. |
| Data analysis. Abstract says sampling weights were used only for descriptive statistics (not for logistic regression). But data analysis paragraph says logistic regression is weighted for the complex sampling design. Which of these was used, and why? Need to be clear when the sampling weights were used, and reasons for not using them for other procedures (if any). Some of the related older literatures are: (A) Gary Solon, Steven J. Haider, Jeffrey M. Wooldridge Solon (2015). What Are We Weighting For? The journal of human resources 50(2): 301-316. (B) Christopher Winship and Larry Radbill (1994). Sampling weights and regression analysis. sociological methods & research. 23(2):230-25 | **Comment accepted and addressed.**   - The statement under the abstract section was not complete because all statistical analyses presented in the manuscript were produced using sampling weights. In the updated abstract, I have indicated that logistic regressions applied sampling weights as well. |
| Table3 refers to a full model and a reduced model. I assume full model is the multivariable model that included all the variables identified from unreported individual univariable logistic models (Identified as Pvalue less than or equal to 0.1). Why OR from this multivariable model is labelled as uOR? What is reduced model? Data analysis section has no mention of such a model. | **Comment accepted and addressed.**   - The labelling of the table’s columns was not properly done. It has been updated to reflect the meaning of the data reported. In the methods section, I have also explained how the full and reduced models were constructed. |
| I have some confusion with Table1 contents. I assume it has a formatting issue. Contents in subheadings rows are not aligned with columns. So, I assume the first column is for males, second column is for females, and 3rd column is for total. If so why percentages for the total is larger than those for males and females? Same issue in table2 too. Also, I noted the percentages for Perpetrators of emotional violence for total sample column sum to 100. Does that mean no one had multiple perpetrators? | **Comment accepted and addressed.**   - The table formatting has been reviewed and confusion removed. |
| Miner comments. Spellings: ow-income to be low income. Spellings: “To obtained the adjusted logistic regression models,” should be “To obtain the adjusted logistic regression models, …”. Older heads of household is 31+ years (not 51+ years)? Some subheading numbers in the manuscript not matching to their main headings Please remove table3 footnote and associated asterisks, they are redundant. | **Comment accepted and addressed.**   - Spelling mistakes checked and corrected |
| **Comments to the Author** |  |
| 1. Is the manuscript technically sound, and do the data support the conclusions?  The manuscript must describe a technically sound piece of scientific research with data that supports the conclusions. Experiments must have been conducted rigorously, with appropriate controls, replication, and sample sizes. The conclusions must be drawn appropriately based on the data presented.  Reviewer #1: Yes  Reviewer #2: Partly | **Comments taken.**   - The table formatting has been reviewed and confusion removed. I took of the mixture appreciation by both reviewers and made necessary changes and write-ups to make the manuscript more technically sound |
| 2. Has the statistical analysis been performed appropriately and rigorously?  Reviewer #1: No  Reviewer #2: Yes | **Comment taken.**   - In the initial submission, tables of results were not properly presented, creating confusion. I have now included updated tables. In the methodology, I have also clearly explained how analyses were done and what is reported in tables. |
| 3. Have the authors made all data underlying the findings in their manuscript fully available?  The [PLOS Data policy](http://www.plosone.org/static/policies.action#sharing) requires authors to make all data underlying the findings described in their manuscript fully available without restriction, with rare exception (please refer to the Data Availability Statement in the manuscript PDF file). The data should be provided as part of the manuscript or its supporting information, or deposited to a public repository. For example, in addition to summary statistics, the data points behind means, medians and variance measures should be available. If there are restrictions on publicly sharing data—e.g. participant privacy or use of data from a third party—those must be specified.  Reviewer #1: No  Reviewer #2: Yes | **Comment taken**   - The dataset used to perform analyses presented in this manuscript is available upon request to Rwanda Biomedical Center (email: [info@rbc.gov.rw](mailto:info@rbc.gov.rw)) or the Principal Investigator ( Yvonne Kayiteshonga, email: [yvonne.kayiteshonga@rbc.gov.rw](mailto:yvonne.kayiteshonga@rbc.gov.rw) ) |
| 4. Is the manuscript presented in an intelligible fashion and written in standard English?  PLOS ONE does not copyedit accepted manuscripts, so the language in submitted articles must be clear, correct, and unambiguous. Any typographical or grammatical errors should be corrected at revision, so please note any specific errors here.  Reviewer #1: No  Reviewer #2: Yes | **Comment taken**   - While working on the reviewers’ comments, I have taken enough time to improve the write-up and the quality of the language of the manuscript. |
| **Reviewer #1: Reviewer comments** |  |
| Comment 1: 1. TITLE: The title is not scientifically written. It needs to be revised. | **Comment accepted and addressed.**   - I proposed a new Title: *Correlates of Emotional Violence against Children in Rwanda: Findings from a Cross-Sectional National Survey* |
| Comment 2: 2. ABSTRACT: a) The aim needs to be revised. It is not scientifically written. b) Methodology has to mention the elements included as measuring parameters but without details. c) No need for details of methodology in the abstract. Only main points. It should not include the type of statistical analysis. d) Methodology is lacking the study design. | **Comment accepted and addressed.**   - The entire abstract has been rewritten. |
| Comment 3: 3. INTRODUCTION: a) The text of the introduction does not seem to have coherence and integrity. The content is scattered. It should be concise and targeted to the aim. b) Newest Global/ Regional/ Rwanda prevalence of violence against children should be stated. c) The introduction lacks literature on emotional violence against children. d) Risk factors associated with emotional violence against children should be clearly stated. e) The current situation of other developing and developed countries should also be added. f) Explaining why this topic was chosen for analysis in this article is not well written. The benefits of conducting the study to the community should be explained. g) The aim should be clearly stated. | **Comment accepted and addressed.**   - The entire introduction has been rewritten, taking into account all points raised. |
| Comment 4: 4. METHODS: a) The methods will be better if structured in this way: type of the study, study setting, study participants, study tools, sample size, data analysis, and ethical considerations. b) Type of the study should be added. c) Sample size should be mentioned. d) The characteristics of the study participants should be mentioned as inclusion criteria and exclusion criteria (if any) e) The head of household age was recoded into two levels: younger heads of household (less than 30 years) and older heads of household (51 years and more), what about the heads of household aged 30-51 years. f) Were the samples normal or not? g) There was no clear mention of the questionnaire(s) used. h) What literature was reviewed to develop the questionnaire? The authors do not provide any references. Was it piloted to assess its internal consistencies? Was it validated? Additional explanations about the validity and reliability of the questionnaire should be added - explained step by step. (Content and structure validity). i) It is advisable to include the questions as per each domain in the methodology. How to score the questionnaire, specify the cut points in the questionnaire? j) How long did it take to complete each questionnaire? Mention the number of questions in the questionnaire. Did the participant answer it themselves or with assistance from researcher? k) What strategy was devised to increase the accuracy of the study and the accuracy of the answers? What types of technique the authors used to keep/control the data quality? l) Authors should include a reference for using the stated formula in calculating the sample size. Furthermore, the basis of sample size calculation should be mentioned to know the confidence level and the margin of error. How did you determine the sample size? Please give a justification for the sample size. m) How did the authors measure their outcome variables? What types of data (quantitative and/or qualitative) were used? n) Tests of significance for each type of data should be added. Clearly state for which variables each test was performed. o) P-values of less than 0.05 are the usual threshold used to determine significance. p) Please describe more clearly the variable selection. Which variables were considered for univariate analysis? Which variables were included for further modelling based on the univariate analysis? Which kind of modelling was used for the further analysis? q) How did the authors describe household wealth index as highest, middle, and the lower wealth indexes. Could they specify the cutoff points for that. r) Ethical considerations: informed consent form and confidentiality in this study need to be mentioned in this section too. | **Comment accepted and addressed.**   - The entire section methods has been rewritten, taking into account points raised and advices provided. |
| Comment 5: 5. RESULTS: a) In the result section, it is advisable to add test of significant in each table from table 1 and 2. b) Please provide more information about the regression (selection process, selected models). Variables entered in the logistic regression should be mentioned. c) Age was not included in the final regression model; age might contribute to EV. d) No need to conduct univariate logistic regression because it doesn’t consider the confounding variables. e) What was the criteria to pass your variables from univariate to multivariate logistic regression? f) Titles of the tables has to be improved to be more specific. g) Any abbreviations should be mentioned below the tables as CI, aOR, and uOR in table 3. h) It is advisable to combine tables 1 and 2 in one table. i) In table 1: the name of each column header is incomplete. | **Comment accepted and addressed.**  Some of the points raised here have been addressed in the methods sections and inconsistencies in the tables were cleared.   - To add test of significance in each table from table 1 and 2   - Since reported data are nationally representative of children aged 13-17 by gender, I think that reporting confidence intervals is enough. - It is advisable to combine tables 1 and 2 in one table.   - I prefer not to combine both tables due to the objectives of my study |
| Comment 6: 6. DISCUSSION: a) It is advisable to explain the study objective at the beginning of the discussion. b) Please start the discussion with a short summary of the study and the main findings. c) There were no explanations for prevalence rates in different area. The manuscript could be greatly strengthened if the authors could provide highlight on the prevalence of EV against children in other developing and developed countries with similar context. d) The discussion is very short, and the authors have not done a good job of connecting their findings to the literature and interpreting their findings. Discuss by using the scientific reasoning the predictors/ correlates of EV against children in other developing and developed countries with similar context. Compare the findings of the study with other findings and state the reasons for the strengths and weaknesses in each section. e) The discussion can be further strengthened by adding more relevant and recent references. | **Comment accepted and addressed.**   - The Discussion section has been rewritten considering the pieces of advice provided. |
| Comment 7: 7. CONCLUSION: Mention factors separately (positively and negatively associated). | **Comment accepted and addressed.**   - The conclusion section has been reorganized considering the pieces of advice provided |
| Comment 8: 8. STRENGTHS AND LIMITATIONS: a) Please analyze the strengths of the study. b) How the limitations affect the generalizability of the findings. | **Comment accepted and addressed.**   - A paragraph on the study’s strengths has been added. |
| Comment 9: 9. REFERENCES: Please revise reference no. 30 | **Comment accepted and addressed.**   - The reference has been edited. |
| **Reviewer #2:** |  |
| Unfortunately, the research lacks originality. There is substantial amount of literature on this topic from Rwanda settings. Different population groups (age and gender) have been studied. Examples include:  Pierre NJ, Claire MM. Effect of child abuse on students’ academic performance in public secondary schools in Rwanda. Journal of Education. 2021 May 28;4(2).  Blanchette I, Rutembesa E, Habimana E, Caparos S. Long-term cognitive correlates of exposure to trauma: Evidence from Rwanda. Psychological trauma: theory, research, practice, and policy. 2019 Feb;11(2):147.  Bahati C, Izabayo J, Munezero P, Niyonsenga J, Mutesa L. Trends and correlates of intimate partner violence (IPV) victimization in Rwanda: results from the 2015 and 2020 Rwanda Demographic Health Survey (RDHS 2015 and 2020). BMC women's health. 2022 Dec;22(1):1-3.  Thomson DR, Bah AB, Rubanzana WG, Mutesa L. Correlates of intimate partner violence against women during a time of rapid social transition in Rwanda: analysis of the 2005 and 2010 demographic and health surveys. BMC women's health. 2015 Dec;15(1):1-3.  The manuscript adds very little to existing knowledge base in this area. | **Comment not accepted.**   - While this study cannot pretend to explain everything related to the prevalence of emotional violence against children and associated factors in Rwanda, its uniqueness is that it is based on data from a first nationally representative survey that was solely targeting children and youth. I am aware of all studies mentioned as examples in Rwanda, but their scope and design are different from the scope and objectives of my study, and the survey on which it is based. In my understanding, my manuscript is just another point of view on child maltreatment and emotional violence against children in Rwanda. |
