# Supplementary material for: Correlates of emotional violence against children in Rwanda: Findings from a cross-sectional national survey
Source: PLoS One. 2023 Jun 13;18(6):e0286788. doi: 10.1371/journal.pone.0286788 (PMC10263359; doi:10.1371/journal.pone.0286788)
Supplement: Attachment — Submitted filename: Response to Reviewers22012023.docx [file pone.0286788.s004.docx]

Dear Editor,

I am very thankful to you and the reviewers for taking the time and effort to review my manuscripts. I have no doubt that their comments have been critical in improving my manuscript's quality and content. May God Bless you all you. Below is my feedback on comments I received from you on 13 Dec 2022.

| **Additional Editor Comments** | **Authors response** |
| --- | --- |
| Authors reported in the introduction “… the prevalence of EV among children aged 13-17 by a parent, caregiver or adult relative in the past 12 months ranged from ten to over forty percent. The lowest prevalence was reported in Lesotho, with 6.9% of EV in female children and 3.8% of EV in male children [16]. The highest prevalence was reported in Uganda: 22.2% in girls and 22.8% in boys”. These two sentences contradictory to each other. Also the last sentence in the same paragraph is a repetition. | **Comments accepted and addressed**  The paragraph has been rewritten and reorganised to make it more coherent. |
| Surveys were conducted by trained interviewers; I assume the surveys were face-to-face. Why the questionnaires and other survey tools were translated into Kinyarwanda, and back-translated into English? | **Comments accepted and addressed**  Most respondents were native speakers of Kinyarwanda, which explains why there was a dire need to use the language respondents would be more comfortable with. Back translation into English was done to crosscheck if the translation from English into Kinyarwanda was done properly. I have dropped that statement “back translated” to avoid confusion for readers. Back translation was solely done in the interest of finetuning the translation from English into Kinyarwanda. Regarding trained interviewers, they were trained on all components of the survey to ensure that they could collect quality data and abide by the survey standards. |
| tables 1 and 2. I know percentages are weighted, therefore simple arithmetic not working, But how can prevalence of EV become larger for overall (ie; all children) than for males and females across all dimensions? I asked this in the last review too. | **Comments accepted and addressed**  VAC surveys are generally designed to compare violence against children in males and females. Their samples are split. Thus, the reported prevalence of EV becomes larger overall (all children) than for males and females across all dimensions because it is a total of what is observed in females and males.  It should also be noted that sampling weights applied to VAC surveys are intended to correct for unequal probability of selection and adjust for non-response to produce results representative of the national population of male and female children. In the case of the Rwanda VAC, the data set was weighted to obtain parameters representing the total population of male and female children aged 13-17 in Rwanda. A two-step weighting procedure was applied, incorporating: (Step 1) computation of base weight for each sample respondent; and (Step 2) adjustment of the base weights for non-response. Reported “all children” results are the total weighted results for male and female children. Since reported results are disaggregated by gender, a binary concept, I suggest dropping reported results for “all children” because it looks redundant. |
| Table1. "percentage not presented due to unstable estimate". I can imagine situations that can arise this situation. But I can't see why this occur here. For example, taking incidence of EV among females, n=42. Of them if 16.3% (about 7 females) experience it few times, and 20.7% (about 9 females) experience it many times, the balance of about 26 (I said "about" because can change due to weighting for study design) should experience it only once. I can’t think of reasons for 26 out of 42 percentage estimate becomes unstable even if estimator is a weighted one. | **Comments accepted and addressed**  Results of the data analysis presented in table one showed there were zero observations for some variables in female children. I have removed the confusing statement “Unstable” and just reported data (0) as per the results. |
| Table1. Perpetrators of EV. Percentages in 'all children' column sum to 100. Does this say none of the children had more than one type of perpetrator (ie, not counted under multiple perpetrator groups)? I assume it should be the case if it was used as a categorical variable in logistic regression (which is unclear to me from the methods or results). I asked the same question in last review too. Similarly, sums of the percentages in each of the other two columns are much lower to 100%. How can that be explained if all perpetrators belong to one of the listed groups? These type of apparent discrepancies have to be explained to strengthen the trustworthiness of the results. | **Comments accepted and addressed**  The reason why Perpetrators of EV percentages in the 'all children' column sum to 100 is how the question on perpetrators was asked. The question about perpetrators came after two questions which asked: *How old were you the last time this happened?* The question about perpetrators was a follow-up question to the previous question and asked:   - *The parent, adult caregiver or other adult relatives who did this to you the last time, what was the person's relationship to you*?   I have redone analyses on perpetrators and included all responses that respondents provided, without recording and merging some of them as previously done. |
| Please re-check for the typos. Example “Poverty and financial hardship, family violence, unwanted pregnancy, lack of social support, etc. [60,61] are some known behind child abandonment”. | **Comments accepted and addressed**  I rechecked the typos across the manuscript. |
| I noted some variables in tables 1 and 2 are not in table3 (in unadjusted form either). E.g.: Perpetrators of EV variable. How you decided what variables initially included in multivariable model building? | **Comments accepted and addressed**  Some variables in tables 1 and 2 were not included in the multivariable model building (Table 3) because of their collinearity and their effects on the sample decrease. For example, “perpetrators of EV” was dropped from the multivariable analyses because it is about only people who reported EV, and its inclusion in the multivariable analyses decreased the sample size significantly. |
