# Supplementary material for: Correlates of emotional violence against children in Rwanda: Findings from a cross-sectional national survey
Source: PLoS One. 2023 Jun 13;18(6):e0286788. doi: 10.1371/journal.pone.0286788 (PMC10263359; doi:10.1371/journal.pone.0286788)
Supplement: Attachment — Submitted filename: Response to Reviewers28012023.docx [file pone.0286788.s005.docx]

Dear Editor,

I am very thankful to you and the reviewers for taking the time and effort to again review my manuscripts. I do not doubt that their comments are critical in improving my manuscript's quality and content. May God Bless you all you. Below is my feedback on comments I received from you on 27 January 2023.

| **Additional Editor Comments** | **Authors response** |
| --- | --- |
| As a response to my comment in previous review, authors say, prevalence of EV become larger for overall (ie; all children) than for males and females across all dimensions because “ …. it is the total for what is observed for females and males”. I disagree because prevalence for males and females cannot be summed in this way. This is not visible now because “all children” column has been removed from tables 2 and 3 of R2 version (tables 1 and 2 in previous version). | **Comments accepted and addressed**  To avoid confusion and for purposes of transparency, I have now reported all data on the prevalence. In previous tables, based on my experiences with other Journals regarding data on outcomes or prevalence, I had omitted those who did not experience EV. I have reintroduced the column of “all children” in tables two and three for your consideration. |
| Authors results lacks internal consistency; they are contradictory to themselves at some places. For example, authors haven’t explicitly reported how many children had experienced the outcome. According to table2, numbers are 78 males and 42 females. Using simple arithmetic (ignoring weightings for rough estimation) these become 12.6% males out of 618 and 8.5% females out of 492. Corresponding %s are reported as 8.9% for males and 5.2% for females. I assume differences could be due to weighting. But, why column percentages do not sum to near 100% whenever they should be? For example, every person who experienced EV should experience it at once or few times or more than few times. But I noted those percentages in table2 sum to 63% for males and 37% for females. Similarly, every person who experienced EV should have one or more perpetrators. However I noted perpetrators percentages in table2 sum to 62% for males and 37.9% for females. Also, number of children who experienced EV reported here differently as 97 males and 55 females. I assume missing values (and possibility of multiple responses for the survey questions) must also have played a role here; authors need to pay attention to them in the analysis. | **Comments accepted and addressed**  Most concerns raised under this section are addressed in my above response to the initial comment; and by the fact that I reintroduced the column for all children for your reference.  Regarding your comment on perpetrators, I had also explained it in my previous revisions. The reason why the perpetrators of EV percentages in the 'all children' column summed to 100 was how the question on perpetrators was asked. The question about perpetrators came after a question which asked: *How old were you the last time this happened?* The question about perpetrators was a follow-up question to the previous question and asked:   - *The parent, adult caregiver or other adult relatives who did this to you the last time, what was the person's relationship to you*?   Thus, it summed up to 100% because children were only asked to refer to the latest time when they experienced EV as a reference point in time. You will also notice that, in my current revisions, I decided to report all responses reported by children without regrouping them as I had done in my previous submission. Given how the question on perpetrators was asked, there were no missing values and the possibility of multiple responses for the survey questions. |
| This is related to the “unstable” estimate mentioned in the previous version which were replaced by zero in current submission. Does the zero observations in data is compatible with the rest of the data? What are the reasons for zero observations? Are they due to not having such people, or response being ‘missing’, or response being ‘not applicable”? Have authors done a proper data cleaning to assess these prior to data analysis? Please amend the article to remove above listed (and other) apparent inconsistencies within results. Impossible to accept an article with apparent contradictions. | **Comments addressed**  To remove confusion around statements “unstable” and “zero observations, I decided to report “n” in table 2. On data cleaning, Rwanda Biomedical Center gave us a cleaned database with all weights and procedures of applying them when conducting analyses. They dealt with all the missing values. Thus, in the reported data, zero observations should not be confused with missing data/values. |
