# Supplementary material for: Correlates of emotional violence against children in Rwanda: Findings from a cross-sectional national survey
Source: PLoS One. 2023 Jun 13;18(6):e0286788. doi: 10.1371/journal.pone.0286788 (PMC10263359; doi:10.1371/journal.pone.0286788)
Supplement: Attachment — Submitted filename: Response to Reviewers08032023.docx [file pone.0286788.s006.docx]

Dear Editor,

Below is my comment on your latest feedback on my manuscript submitted on January 28, 2023.

| **Additional Editor Comments** | **Authors response** |
| --- | --- |
| Authors tried to respond to the issue of internal inconsistency in reporting findings. But the issue is still remains. For example, out of 618 boys included in the study, 101 experienced EV. 101/618=16.3%. But it is reported in table2 as EV prevalence of 8.87% . Similarly, of the 492 girls 55 experienced EV. 55/492=11.2%. But it is reported in table2 as 5.17% EV prevalence. For all children, 156 out of 954 experienced EV, this is reported as 14.04% EV. This last result seems correct because 156/1110=14.05%. However, authors say, prevalences in two genders were summed to get the overall prevalence (ie; 8.87+5.17=14.04) which is totally incorrect logic. This is an example only. There are numerous places in the manuscript with incompatible reporting, and incorrect calculations. Therefore I can't trust these calculations presented, nor the conclusions based on them. | **Comments not accepted**  Unfortunately, we do not yet have a common understanding that the data I reported in this manuscript were weighted to represent a population of male and female children aged 13-17 in Rwanda. We cannot compare weighted reported percentages with percentages calculated from unweighted raw data from the survey. To avoid the persisting confusion between the data I reported and the results of the arithmetic calculations that you suggest based on “n” introduced in table 2, I suggest that we drop the “n” columns in the manuscript version I submitted on January 28, 2023, and keep the version I submitted on January 22, 2023. |
