# Supplementary material for: Correlates of emotional violence against children in Rwanda: Findings from a cross-sectional national survey
Source: PLoS One. 2023 Jun 13;18(6):e0286788. doi: 10.1371/journal.pone.0286788 (PMC10263359; doi:10.1371/journal.pone.0286788)
Supplement: Attachment — Submitted filename: Response to Reviewers24042023.docx [file pone.0286788.s007.docx]

Dear Editor,

I am very thankful to you and the reviewers for taking the time and effort to review my manuscripts. I do not doubt that their comments have improved my manuscript's quality and content. May God Bless you all you. Below is my feedback on comments I received from you on April 20, 2023.

| **Additional Editor Comments** | **Authors response** |
| --- | --- |
| 1. Authors may add CI after the odds ratio in the abstract to see the significance of the results. | **The comment is accepted**. CIs have been added after the odds ratios reported in the abstract. |
| 2. In the sentence “Mothers were responsible for seventeen percent (17.25 %, 95% CI [11.86-24.42]) of EV against male children, and nine percent (8.7 %, 95% CI [4.75-15.49]) of female children (8.7 %, 95% CI [4.75-15.49]) reported EV by their mothers.” Results are repeated. Authors should avoid the repetition of the results. | **The comment is accepted.** The indicated repetition was removed, and the section was rewritten correctly. |
| 3. Authors should maintain the consistency during interpretation of results that were round figured by them. | **The comment is accepted**. The results section has been rewritten in alignment with how results appear in Tables 2 and 3. |
| 4. I do not understand why some factors in tables have different sample sizes as the overall sample size for the current study is 1110. The authors should explain it. | **The comment is accepted**. I have reorganised the presentation of data in the Tables to ensure everything is clear. I have also added some explanatory notes. As shown in Tables 2 and 4, the overall sample size of children on which data analyses were conducted in 1110 (including 618 male and 492 female children in Table 2) and 1073 in Table 4(Logistic regression). In Table 2, however, we also presented data on the incidence and perpetrators of EV from 120 children who reported having experienced emotional violence in the last 12 months before the survey. In this study, while children were also asked about their lifetime experiences of EV, we preferred “Self-reported EV within twelve months before the survey date” as the outcome variable to control for any recall bias. |
| 5. A detailed literature review on predictors of emotional violence would give a clear picture as to why the authors considered them in the analysis. | **The comment is accepted**.  I have explained more about the factors assessed for association with emotional violence. |
| 6. Discussion on why a girl child is less emotionally abused is not justified by the present discussion. In fact, the authors seem to give a completely opposite picture of it. | **The comment is accepted**.  I have revised the discussion statement on why a girl child is less emotionally abused. |
| 7. For a better understanding of it, authors should create a variable “family with more or less son than a daughter or other combination of a different gender to get a clear picture of son preference. | I agree with the suggestion. Unfortunately, the design and current data in the Rwanda violence against children and youth survey cannot allow those computations. Another study is required to assess son preference in Rwanda. |
