# Supplementary material for: Correlates of emotional violence against children in Rwanda: Findings from a cross-sectional national survey
Source: PLoS One. 2023 Jun 13;18(6):e0286788. doi: 10.1371/journal.pone.0286788 (PMC10263359; doi:10.1371/journal.pone.0286788)
Supplement: Attachment — Submitted filename: Reviewer comments PONE-D-22-24606.docx [file pone.0286788.s001.docx]

Reviewer comments

Manuscript Number: PONE-D-22-24606

Title "Correlates of Emotional Violence against Children in Rwanda".

**Generally speaking:**
Thank you for providing me the opportunity to review this manuscript that raises important and interesting issues about correlates of emotional violence against children in one of the developing countries.

**Comment 1:**

1. *TITLE:*

The title is not scientifically written. It needs to be revised.

**Comment 2:**

2. *ABSTRACT:*
a) The aim needs to be revised. It is not scientifically written.
b) Methodology has to mention the elements included as measuring parameter but without details.
c) No need for details of methodology in the abstract. Only main points. It should not include the type of statistical analysis.
d) Methodology is lacking the study design.

**Comment 3:**
*3. INTRODUCTION:*

a) The text of the introduction does not seem to have coherence and integrity. Content is scattered. It should be concise and targeted to the aim.
b) Newest Global/ Regional/ Rwanda prevalence of violence against children should be stated.
c) The introduction lacks literature on emotional violence against children.
d) Risk factors associated with emotional violence against children should be clearly stated.
e) The current situation of other developing and developed countries should also be added.
f) Explaining why this topic was chosen for analysis in this article is not well written. The benefits of conducting the study to the community should be explained.
g) The aim should be clearly stated.

**Comment 4:**
*4. METHODS:*

a) The methods will be better if structured in this way: type of the study, study setting, study participants, study tools, sample size, data analysis, and ethical considerations.
b) Type of the study should be added.
c) Sample size should be mentioned.
d) The characteristics of the study participants should be mentioned as inclusion criteria and exclusion criteria (if any)
e) The head of household age was recoded into two levels: younger heads of household (less than 30 years) and older heads of household (51 years and more), what about the heads of household aged 30-51 years.
f) Were the samples normal or not?
g) There was no clear mention of the questionnaire(s) used.
h) What literature was reviewed to develop the questionnaire? The authors do not provide any references. Was it piloted to assess its internal consistencies? Was it validated? Additional explanations about the validity and reliability of the questionnaire should be added - explained step by step. (Content and structure validity).
i) It is advisable to include the questions as per each domain in the methodology. How to score the questionnaire, specify the cut points in the questionnaire?
j) How long did it take to complete each questionnaire? Mention the number of questions in the questionnaire. Did the participant answer it themselves or with assistance from researcher?
k) What strategy was devised to increase the accuracy of the study and the accuracy of the answers? What types of technique the authors used to keep/control the data quality?
l) Authors should include a reference for using the stated formula in calculating the sample size. Furthermore, the basis of sample size calculation should be mentioned to know the confidence level and the margin of error. How did you determine the sample size? Please give a justification for the sample size.
m) How did the authors measure their outcome variables? What types of data (quantitative and/or qualitative) were used?
n) Tests of significance for each type of data should be added. Clearly state for which variables each test was performed.
o) P-values of less than 0.05 are the usual threshold used to determine significance.
p) Please describe more clearly the variable selection. Which variables were considered for univariate analysis? Which variables were included for further modelling based on the univariate analysis? Which kind of modelling was used for the further analysis?
q) How did the authors describe household wealth index as highest, middle, and the lower wealth indexes. Could they specify the cutoff points for that.
r) Ethical considerations: informed consent form and confidentiality in this study need to be mentioned in this section too.

**Comment 5:**
*5. RESULTS:*

a) In the result section, it is advisable to add test of significant in each table from table 1 and 2.
b) Please provide more information about the regression (selection process, selected models). Variables entered in the logistic regression should be mentioned.
c) Age was not included in the final regression model; age might contribute to EV.
d) No need to conduct univariate logistic regression because it doesn’t consider the confounding variables.
e) What was the criteria to pass your variables from univariate to multivariate logistic regression?
f) Titles of the tables has to be improved to be more specific.
g) Any abbreviations should be mentioned below the tables as CI, aOR, and uOR in table 3.
h) It is advisable to combine tables 1 and 2 in one table.
i) In table 1: the name of each column header is incomplete.

**Comment 6:**

*6. DISCUSSION:*

a) It is advisable to explain the study objective at the beginning of the discussion.
b) Please start the discussion with a short summary of the study and the main findings.
c) There were no explanations for prevalence rates in different area. The manuscript could be greatly strengthened if the authors could provide highlight on the prevalence of EV against children in other developing and developed countries with similar context.
d) The discussion is very short, and the authors have not done a good job of connecting their findings to the literature and interpreting their findings. Discuss by using the scientific reasoning the predictors/ correlates of EV against children in other developing and developed countries with similar context. Compare the findings of the study with other findings and state the reasons for the strengths and weaknesses in each section.
e) The discussion can be further strengthened by adding more relevant and recent references.

**Comment 7:**

*7. CONCLUSION:*
Mention factors separately (positively and negatively associated).

**Comment 8:**

*8. STRENGTHS AND LIMITATIONS:*

a) Please analyze the strengths of the study.
b) How the limitations affect the generalizability of the findings.

**Comment 9:**

*9. REFERENCES:*
Please revise reference no. 30
