# Supplementary material for: Correlates of emotional violence against children in Rwanda: Findings from a cross-sectional national survey
Source: PLoS One. 2023 Jun 13;18(6):e0286788. doi: 10.1371/journal.pone.0286788 (PMC10263359; doi:10.1371/journal.pone.0286788)
Supplement: Attachment — Submitted filename: Reviewer comments PONE-D-22-24606_R1.docx [file pone.0286788.s003.docx]

Reviewer comments

Manuscript Number: PONE-D-22-24606_R1

Title "Correlates of Emotional Violence against Children in Rwanda: Findings from a Cross-Sectional National Survey".

Thank you for providing me the opportunity to re-review this manuscript that raises important issues about correlates of emotional violence against children in Rwanda: findings from a cross-sectional national survey in one of the developing countries.

It seems that all corrections were done.
